# Predictions of Alzheimer’s disease treatment and care costs in European countries

**DOI:** 10.1371/journal.pone.0210958

**Published:** 2019-01-25

**Authors:** Richard Cimler, Petra Maresova, Jitka Kuhnova, Kamil Kuca

**Affiliations:** 1 Faculty of Science, University of Hradec Králové, Hradec Králové, Czech Republic; 2 Faculty of Informatics and Management, University of Hradec Králové, Hradec Králové, Czech Republic; Nathan S Kline Institute, UNITED STATES

## Abstract

**Background:**

Given the increasing lifespan of the elderly and the higher proportion of older people in the global population, the incidence rate of neurodegenerative diseases is increasing. The aim of this study is to evaluate, by means of computer simulations, developments in the costs of treating and caring for people suffering from Alzheimer’s disease (AD) in the EU 28 by 2080, while assuming the introduction of drug administrations at various disease stages.

**Methods:**

Impact analysis leverages a mathematical model that compares five different population development scenarios when introducing different types of drugs to the scenarios but without changing the treatment. Changes in the economic burden are considered as of 2023, when new drugs are expected to enter the market.

**Findings:**

The results of the simulations show that by prolonging the length of a person’s ‘stay’ in the Mild, Moderate, or Severe stage, the total cost of care for all persons with AD will increase by 2080. For individual scenarios, the percentage of patients and costs increased as follows: Mild by one year, by 10.61%; Mild by two years, by 17.73%; Moderate by one year, by 16.79%; Moderate by two years, by 34.88%; and Severe by one year, by 23.79%. The change in cost development when prolonging the stay in the Mild cognitive impairment stage (by lowering the incidence by 10%, 30%, or 50%) reduced the cost (by 4.88%, 16.78% and 32.48%, respectively).

**Interpretation:**

The results unambiguously show that any intervention prolonging a patient’s stay in any stage will incur additional care costs and an increase in the number of persons with AD. Therefore, extending lifespan is important in terms of improving the quality of life of patients, and the introduction of new drugs must consider the additional costs imposed upon society.

## Introduction

In the 1990s, numerous studies [1–5] began to highlight the problem of the increasing number of people with dementia in developed countries. The significant financial burden imposed upon the socioeconomic systems of developed countries by virtue of caring for elderly people was recognized [6–8], especially in the context of demographic progress. [9, 10] The increasing efficiency of health care and preventive measures have resulted in increased expenses toward health and social systems [9–11] that are intended to help people of all age groups.

A number of studies based on mathematical models or on qualified estimates focus on prediction with regard to the development of Alzheimer’s disease (AD) in populations. [12–15] There are also economic burden models related to the treatment and care of those afflicted by the disease. Ikeda [16] examined the economic impacts of the use of Donepezil in the mild and moderate stages of AD, while Gustavsson et al. [17] reviewed health economic modelling across the full AD continuum. The available models have been limited by a lack of natural progression data, and many of the most influential models, at a policy level, are based on either very small or outdated samples. Brown et al. [18] simulated AD-related prevalence, morbidity, and mortality for the 2011–2050 period, together with associated caregiver time and costs, through the use of a dynamic computable general equilibrium model as applied to the Chinese economy; they analysed both economic and noneconomic outcomes. Health and macroeconomic models predict an unfolding in 2011–2050 of a Chinese AD epidemic that will have serious macroeconomic consequences. Suh [19] used an existing Markov model that was adapted to the case of South Korea, to predict long-term outcomes over a five-year time horizon and estimate the cost-effectiveness of galantamine in treating AD. The model structure is informed by a review of the national and international literature on the clinical and cost-effectiveness of galantamine and on the costs and outcomes associated with AD treatment. The main outcome measure used was the cost per quality-adjusted life year gained. Changes in the costs of treatment and care in the case of different time options for introducing new drugs to the market have not been specified for the European population, however. [9] It is therefore difficult to evaluate the development of future expenses related to treating and caring for people with AD.

Therefore, the aim of the current study is to simulate the development of costs related to treating and caring for persons afflicted with AD in the EU 28 by 2080; it does so by assuming the introduction of drugs at different disease stages, and uses various computer simulation methods. The change in the economic burden is considered from 2023, when new drugs are expected to enter the market. [20] The results of previous work [21] concern predictions of the population and number of patients in each AD stage; based on these values, we predict the cost of treating them. The model allows us to determine the detailed numbers and costs for each year in the study period, along with the structure of the healthy and diseased population.

## Methods

### Model

The model used herein is an extension of the one described in previous research. [21, 22] Previous studies have created a prediction model for a population with AD in the European Union. There, the population is divided into two main subpopulations—namely, healthy people and people with AD. The total population was simulated by leveraging data from the Eurostat database. The subpopulation with AD was derived by using the prevalence rate [12], and it was then subtracted from the total population to determine the size of the healthy population. Previous research has projected the number of people with AD in the EU until the year 2080, and a comparison of three different simulation approaches—namely, system dynamics, agent-based modelling, and numerical modelling.

The current study takes a numerical approach. One improvement made by this study is that it considers the various AD stages. The model is expanded by specifying the stages of AD through which the patient moves; the likelihood of death depends on which of these stages the patient is currently experiencing. The transition among individual stages, or dying at a given stage, is understood as the realization of Markov’s four-state chain (i.e. Mild, Moderate, Severe, and Death). Additionally, the likelihood of contracting a given disease depends on the prevalence of that disease. All new patients start in the Mild stage, with probability *r* that corresponds to the prevalence of the disease. For people already afflicted by this disease, the probability of transition to a higher (or possibly even a lower) stage of the disease is calculated each year.

In the model, a Mild cognitive impairment (MCI) is considered in the simulated scenarios. MCI is not modelled as a separate stage of the disease: people with an MCI are included in the healthy population, because in every considered aspect (especially the death rate), they are the same as healthy people. According to [23], MCI is used to describe a state in which there is a cognitive decline, but it is not severe enough to meet the diagnostic criteria for dementia. However, in the simulations, the scenarios focused on improvements in mental health in the MCI stage, which would lead to a decrease in new patients in the Mild stage of AD.

### Data collection

#### Population

Data used to predict the total population of the EU 28 were drawn from the EUROSTAT database—namely, the ‘baseline’ prediction, which starts in 2015 and ends in 2080. From this data, mortality was determined in individual age cohorts; similar to the determination of incidence, the mortality rate of healthy individuals was determined when the program was run for the first time, irrespective of the nature of the population afflicted with AD.

Disease prevalence for age cohort *x* was considered according to [12], in the form

$$r_{x}=0.0142\cdot {\text{e}}^{0.1161x}.$$

On the first run of the program, corresponding incidence rates for each age cohort were found in each year of the simulation. All other simulations had already used the incidence rates determined in a such a way, which are independent of the choice of treatment scenario.

Although incidence relates to the whole of the healthy population, transition to the Mild stage of a disease is considered only from the MCI stage. This reflects the fact that, nowadays, people with MCI are usually not considered part of the healthy population; it also reflects the fact that MCI does not affect a person’s mortality. Every AD patient in the model has passed through the MCI stage [23].

To identify individual stages and the probability of death, we used data from the National Alzheimer’s Coordinating Centre in Great Britain [24], which collected and processed these data. The research sample included 3,852 patients with possible AD who were aged over 50 years. Patients are divided into three groups in accordance with the severity of their disease—namely, Mild, Moderate, or Severe. The probabilities of staying in or of a transitioning among individual stages are listed in Table 1; the probabilities of death in the individual stages are follows: 5.5% for Mild, 21.5% for Moderate, and 48.0% for Severe.

**Table 1:** Probabilities of disease-stage transition, according to National Alzheimer’s Coordinating Centre data [24].

Stage [25]	Staying	Improving by one	Worsening by one	Worsening by two
Mild	77.4%	–	15.8%	1.3%
Moderate	50.1%	7.0%	21.4%	–
Severe	49.2%	2.8%	–	–

The initial distribution of the population of patients into individual disease stages is based on the limit distribution of these stages after the simulation has ended.

#### Costs

Given the lack of uniform evidence on the cost of treating and caring for people suffering from AD in EU countries, only the cost data cited in the study were used. [26] However, since only two stages are considered in the current study, the Moderate-stage costs were divided in line with the cost ratio of the Moderate and Severe stages reported in other studies [27, 28] as average values. The selection of studies was based on the following criteria: costs in EU countries, distribution of direct and indirect costs, linking the costs to the three disease stages (Mild, Moderate, or Severe), and the age of the study published after 2010. The costs for the individual disease stages are shown in Table 2.

**Table 2:** Cost classifications of individual stages, based on disease stage and type of care.

	Informal	Nonmedical	Medical
Mild	1027.00	619.00	280.00
Moderate	1676.64	1432.20	234.36
Severe	2315.36	1977.80	323.64

The costs cited in the studies have a uniform classification, according to the methodology of Michael Drummond [29] for direct and indirect costs. Specifically, informal costs pertain to surveillance and the fulfilment of activities of daily living. Nonmedical costs pertain to the use of daycare, alarm services, home help, and day/night surveillance, while medical costs pertain to inpatient and outpatient hospital visits, physician visits, speech therapist visits, physical therapist visits, nurse visits, and medication use.

We do not consider the costs of treatment or care in relation to MCI, as AD is not diagnosed in the MCI stage. Experts use many diagnostic options, such as neurological examinations, blood sampling, magnetic resonance imaging, and computed tomography. Various self-diagnostics that leverage modern technologies are also considered. The cost range is therefore wide, and their inclusion would derive slightly misleading results. There are also other types of health costs, both direct and indirect, that relate to AD but are not considered here, because the diagnostic criteria for dementia are not met at this stage [23].

### Design

In the model, the total population of the EU 28 is divided into two groups according to prevalence—namely, sick and healthy individuals. Initially, sick individuals are categorized as per their disease stage. At each subsequent simulation step, the proportion of patients who will survive the following year is determined; subsequently, it is determined whether they will remain in the current disease stage or move to a different one. At the same time, the proportion of surviving healthy individuals is determined. At the end of each year of the simulation, the individual cohorts in both populations are assigned an age increased by one year; additionally, new patients are generated by incidence, and they are concurrently removed from the healthy population.

After the simulation finishes, tables showing the numbers of healthy and sick (i.e. by disease stage) individuals are available for each year of the simulation, and for each age cohort. These data are then processed with respect to the patient’s cost at a particular stage.

The aim of this study is to evaluate the effects of introducing the use of a drug that will prolong each patient’s ‘stay’ in a disease stage. Thus, by adjusting the probabilities of transition among the stages, it is possible to simulate, in addition to the basic course, scenarios in which a patient in the Mild or Moderate stage can extend his or her stay there by one or two years. To prolong the stay in the Severe stage, the probability of patient death in this disease stage was modified. Changes to transition probabilities were made in line with a fundamental matrix that considers an absorbing Markov chain. That fundamental matrix contains information about the expected number of steps (e.g. in our simulation, years) for which the chain stays in the specific stage.

Also important—perhaps even most important—is the possibility of delaying the onset of AD symptoms (i.e. the transition from the MCI stage to the Mild stage). Thus, we created three scenarios, wherein we lower the incidence rates by 10%, 30%, or 50%.

The simulation model was created in the Octave program; the data were subsequently processed using R software, which also produced the resulting graphs.

## Results

To compare the costs in the individual scenarios to those in the current situation, we use the model to predict the development of the number of patients and costs when there are no changes to the style of treatment (based on the aforementioned probabilities of staying in individual stages, or transitioning); we also predict respective death rates (Table 3).

**Table 3:** Costs (in billions of €) in selected years of simulation, for individual disease stages and no change in treatment (reference simulations).

	2015	2030	2040	2050	2060	2070	2080
Mild	119.6	222.0	276.0	330.4	368.2	383.9	395.2
Moderate	66.8	119.5	149.4	180.8	204.6	215.8	222.0
Severe	45.6	79.2	99.6	121.3	139.0	148.1	152.5
Overall	232.0	420.8	525.0	632.6	711.8	747.7	769.7

This situation corresponds to the costs that will be incurred in the future, unless the method of treatment changes.

The annual cost pertaining to the Mild stage start at €120 billion in 2015, reaching a maximum of €395 billion at the end of the simulation in 2080. For the Moderate stage, this cost starts at €67 billion and reaches the maximum of €222 billion. For the Severe stage, these costs are €46 billion and €153 billion. The total costs, by disease stage, in the 66-year simulation are as follows: Mild stage, €20.0 trillion; Moderate stage, €11.0 trillion; and Severe stage, €7.4 trillion.

In other scenarios, the likelihood of a transition between individual stages is adjusted, so that the average period during which one stays in a certain stage is longer; this assumption consequently reflects in the treatment and care costs that are incurred. In all the simulation scenarios, the introduction of a drug in 2023 is assumed, which reflects the current progression in developing new drugs and introducing them to the market [20].

### Cost development when prolonging stay in the Mild stage of the disease

In the Mild stage, it is assumed that a drug or method is applied that will prolong the stay in that stage by one or two years, on average. For one year, the probabilities were adjusted as follows: the probability of staying in the Mild stage increased to 81.4%, compared to the original value of 77.4% (see Table 1); transition to the Moderate stage dropped to 12.1%; and transition to the Severe stage dropped to 1%. For a two-year extension, the probability of staying in the Mild stage changed to 84.4%; transition to the Moderate stage dropped to 9.2%; and transition to the Severe stage dropped to 0.9%. The results are shown in Table 4.

**Table 4:** Change in cost development when prolonging the stay in the Mild stage by one or two years.

**Costs from staying in the Mild stage for one more year**						
	2030	2040	2050	2060	2070	2080
Mild	30.3	48.0	60.4	70.3	76.1	79.0
	113.66%	117.37%	118.29%	119.09%	119.81%	120.00%
Moderate	−5.8	−0.2	1.5	3.1	4.6	5.3
	95.15%	99.86%	100.81%	101.49%	102.15%	102.40%
Severe	−8.0	−4.5	−4.1	−3.8	−3.1	-2.7
	89.86%	95.47%	96.62%	97.27%	97.91%	98.22%
Overall	16.5	43.2	57.8	69.6	77.6	81.7
	103.92%	108.24%	109.14%	109.77%	110.38%	110.61%
**Costs from staying in the Mild stage for two more years**						
	2030	2040	2050	2060	2070	2080
Mild	55.8	92.2	118.0	138.2	150.4	156.8
	125.13%	133.41%	135.70%	137.53%	139.17%	139.67%
Moderate	−18.4	−11.0	−9.8	−8.4	−6.3	−5.3
	84.59%	92.64%	94.60%	95.89%	97.10%	97.61%
Severe	−18.9	−15.0	−15.6	−16.1	−15.4	−15.0
	76.19%	84.89%	87.16%	88.40%	89.59%	90.19%
Overall	18.5	66.2	92.6	113.7	128.7	136.5
	104.40%	112.61%	114.64%	115.97%	117.21%	117.73%

Staying in the Mild stage for one more year will increase costs in all predicted periods; for the year 2080, it is estimated that costs associated with patients in this stage will increase by almost 20%. This increase is because more people will be treated and cared for, on account of both demographic developments and prolonged life thanks to treatment. Costs associated with the Moderate and Severe stages will remain at approximately the same levels (change ±2%). This fact reflects in subsequent stages (in the form of savings in 2030 in the Moderate and Severe stages), which patients will arrive at later. For the Severe stage, there will be an approximately 11% cost savings in 2080; however, given the fact that the costs associated with the Moderate and Severe stages are, in absolute values, significantly higher than those in the Mild stage, in the overall comparison (regardless of stage), there is a slight increase in costs (i.e. by 110.6% in 2080). When extending the stay in the Mild stage by two years, there are significantly greater savings for both the Moderate stage (less than 2.39% in 2080) and the Severe stage (by almost 9.81% in 2080). However, overall, the cost for all the stages will increase by almost 18% in 2080, compared to the simulation in which no treatment is introduced.

**Fig 1:**
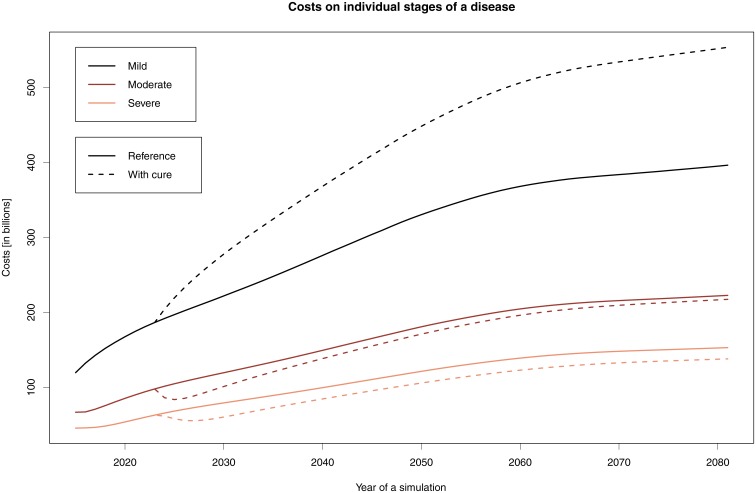
Cost development when staying in the Mild stage for two more years. The graph shows the costs associated with the individual stages: solid lines indicate the reference simulations, while dashed lines indicate the simulations where the stay in the Mild stage is prolonged for two years.

Fig 1 illustrates cost developments in the individual stages following the introduction of a drug that will prolong by two years a patient’s stay in the Mild stage. The intermittent curves show the change in costs following the initiation of treatment: costs will increase significantly in the Mild stage and be lower in the later stages.

### Cost development when prolonging stay in the Moderate stage of the disease

To simulate staying in the Moderate stage for one more year, the probabilities were adjusted as follows. The probability of staying in the Moderate stage increased to 70%; compare this to the value of 50.1% from Table 1. The probability of transitioning to the Mild and Severe stages decreased to 5% and 4%, respectively. For the two-year extension, the probability of staying in the Moderate stage increased to 77%; the probability of transitioning to the Mild and Severe stages decreased to 4% and 0.4%, respectively.

In the Moderate stage alone, when staying for one more year, the costs will increase by 61.29%; consequently, there will be a 4.66% savings in the Severe stage for the corresponding year 2080 (see Table 5). Th cost development when staying in the Moderate stage of the disease for two more years results in savings only in the Severe stage—namely, 6.79% in 2080. There is also a moderate cost increase in the Mild stage, given the fact that if the period of staying in the Moderate stage were extended, the number of patients who would improve would also increase. Therefore, in the Mild stage, in addition to the number of patients in the reference model, there will also be more patients improving from the Moderate stage. Therefore, there is a slight increase in costs even in the Mild stage. Overall, there is a 34.88% increase in 2080 when prolonging the stay in the Moderate stage by two years, compared to the simulation in which there were no treatment changes.

**Table 5:** Change in cost development when prolonging the stay in the Moderate stage by one or two years.

**Costs of staying in the Moderate stage for one more year**						
	2030	2040	2050	2060	2070	2080
Mild	−2.6	−1.7	−1.6	−1.6	−1.4	−1.3
	98.83%	99.40%	99.50%	99.56%	99.63%	99.67%
Moderate	61.7	85.0	104.9	121.8	131.5	136.1
	151.60%	156.90%	158.03%	159.52%	160.95%	161.29%
Severe	−8.8	−6.0	−6.5	−6.5	−5.8	−5.6
	88.90%	93.94%	94.60%	95.30%	96.05%	96.34%
Overall	50.3	77.3	96.7	113.6	124.2	129.2
	111.95%	114.72%	115.29%	115.96%	116.61%	116.79%
**Costs of staying in the Moderate stage for two more years**						
	2030	2040	2050	2060	2070	2080
Mild	−3.9	−1.5	−.7	−.1	.6	1.0
	98.27%	99.47%	99.79%	99.96%	100.15%	100.27%
Moderate	110.3	166.3	208.8	244.8	266.9	277.8
	192.28%	211.30%	215.51%	219.64%	223.73%	225.11%
Severe	−17.8	−12.1	−12.5	−12.4	−11.1	−10.4
	77.54%	87.87%	89.69%	91.06%	92.52%	93.21%
Overall	88.6	152.7	195.7	232.3	256.4	268.5
	121.07%	129.09%	130.93%	132.63%	134.30%	134.88%

**Fig 2:**
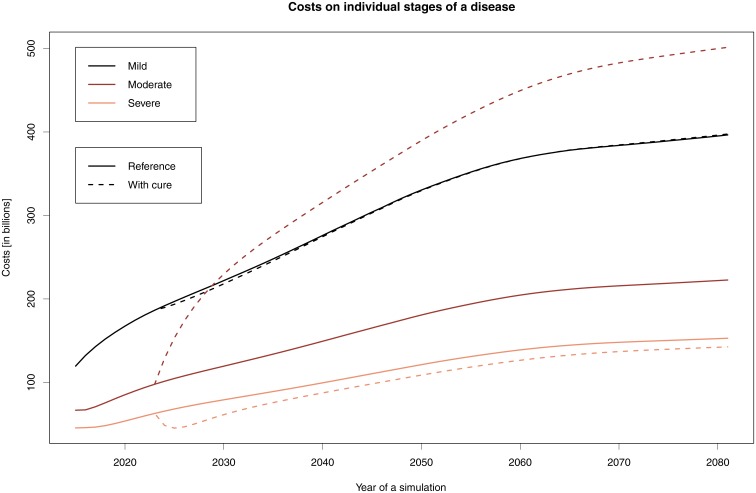
Cost development when staying in the Moderate stage for two more years. The graph shows the costs associated with the individual stages: solid lines indicate the reference simulations, while dashed lines indicate the simulations where the stay in the Moderate stage is prolonged for two years.

Fig 2 shows changes in treatment costs in individual stages after staying in the Moderate stage for two years, compared to the reference simulation.

### Cost development when prolonging stay in the Severe stage of the disease

In this scenario, the probability of death changed; that is because, in addition to the unlikely transition to the Moderate stage, this was the only possible transition for the patient. The likelihood of dying in the Severe stage was reduced from the original 48%, to 20%. In this stage, only a one-year extension was simulated; if a stay longer than one year were to be modelled, the probability of death would be lower than that in the Moderate stage, and this result would not align with reality.

Within this scenario, no more savings arose: there was only an increase in the health and social costs of treating and caring for afflicted individuals (i.e. by 23.79% overall); see Table 6. Attempts to stay in this stage longer would likely not contribute to any improvements in quality of life.

**Table 6:** Change in cost development when prolonging the stay in the Severe stage by one year.

Costs of staying in the Severe stage for one more year						
	2030	2040	2050	2060	2070	2080
Mild	−0.3	−0.4	−0.5	−0.6	−0.7	−0.7
	99.88%	99.85%	99.84%	99.83%	99.82%	99.82%
Moderate	−2.1	−2.6	−3.2	−3.7	−4.0	−4.1
	98.20%	98.24%	98.22%	98.19%	98.15%	98.15%
Severe	75.4	111.9	139.5	164.4	180.5	188.0
	195.16%	212.35%	214.96%	218.23%	221.87%	223.25%
Overall	73.0	108.8	135.7	160.0	175.8	183.1
	117.35%	120.73%	121.46%	122.48%	123.51%	123.79%

### Cost development when prolonging the MCI stage

The most recent model describes a scenario where preventive measures would be applied with respect to AD in a healthy population aged 65 and over. For example, the area diagnosis of healthy people can be considered, followed by the treatment of people with MCI, or area prevention (which relates entirely to the outcomes of research and development that is currently under way). However, these approaches to AD would result in a subsequent reduction in AD incidence. By lowering the incidence rate by 10%, 30%, or 50% (similar to [30]), we simulate the prolongation of the stay of ‘not-yet patients’ in the MCI stage. These scenarios show that this approach results in across-the-board savings.

**Fig 3:**
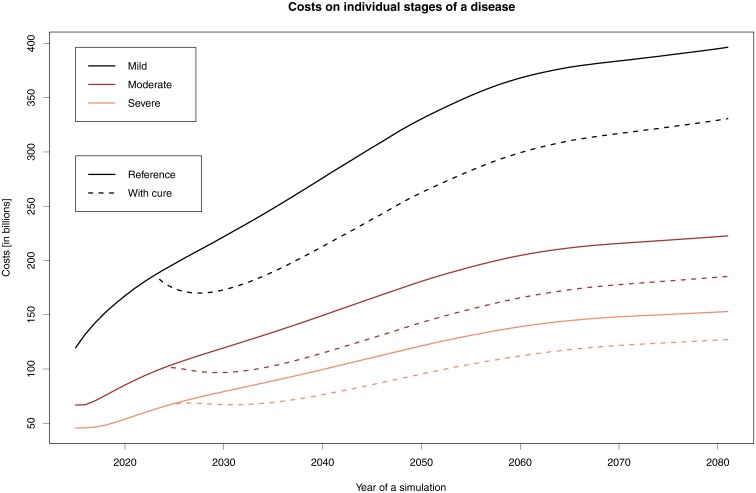
Cost development when prolonging the MCI stage of AD by 30%. The graph shows the costs associated with the individual stages: solid lines indicate the reference simulations, while dashed lines indicate the simulations where the stay in the Moderate stage is prolonged by two years.

Both Table 7 and Fig 3 clearly show savings enjoyed by the overall healthcare system. These savings speak to the only unambiguously cost-effective solution in this area, which in turn points to the meaningfulness of research and development in this AD stage. For example, should treatment or prevention lead to a 30% decrease in the number of AD cases, cost savings given the current prices and demographic structure would rise to 17%; if that 30% figure were to instead increase to 50%, savings would increase to about 33% (see Table 7).

**Table 7:** Change in cost development when prolonging the stay in the MCI stage by lowering the incidence by 10%, 30%, or 50%.

**Costs of staying in the MCI stage by lowering the incidence by 10%**						
	2030	2040	2050	2060	2070	2080
Mild	−15.9	−19.7	−20.7	−20.5	−19.6	−19.1
	92.83%	92.85%	93.75%	94.43%	94.89%	95.16%
Moderate	−7.4	−10.9	−11.6	−11.7	−11.2	−10.9
	93.78%	92.70%	93.57%	94.29%	94.82%	95.10%
Severe	−4.0	−7.3	−8.0	−8.1	−7.8	−7.5
	95.00%	92.63%	93.42%	94.18%	94.76%	95.06%
Overall	−27.3	−38.0	−40.3	−40.3	−38.5	−37.5
	93.51%	92.76%	93.63%	94.34%	94.84%	95.12%
**Costs of staying in the MCI stage by lowering the incidence by 30%**						
	2030	2040	2050	2060	2070	2080
Mild	−49.0	−63.1	−67.9	−68.8	−66.9	−65.9
	77.91%	77.14%	79.46%	81.31%	82.59%	83.33%
Moderate	−22.8	−34.6	−38.0	−39.0	−38.0	−37.4
	80.96%	76.82%	79.00%	80.93%	82.39%	83.16%
Severe	−12.1	−23.2	−26.0	−26.9	−26.3	−25.9
	84.78%	76.71%	78.61%	80.63%	82.21%	83.04%
Overall	−83.9	−120.9	−131.8	−134.8	−131.2	−129.1
	80.07%	76.97%	79.17%	81.07%	82.45%	83.22%
**Costs of staying in the MCI stage by lowering the incidence by 50%**						
	2030	2040	2050	2060	2070	2080
Mild	−84.1	−112.4	−124.7	−129.6	−128.1	−127.8
	62.13%	59.27%	62.26%	64.81%	66.62%	67.67%
Moderate	−38.7	−61.3	−69.4	−73.1	−72.6	−72.3
	67.63%	58.98%	61.64%	64.28%	66.34%	67.43%
Severe	−20.4	−40.8	−47.2	−50.2	−50.2	−49.9
	74.25%	59.03%	61.12%	63.87%	66.08%	67.26%
Overall	−143.2	−214.5	−241.2	−252.9	−251.0	−250.0
	65.98%	59.14%	61.86%	64.47%	66.43%	67.52%

## Discussion

The objective of the current study is to simulate cost developments associated with treating and caring for people suffering from AD in the EU 28 by 2080, while assuming the introduction of drugs at various stages of disease onset.

**Fig 4:**
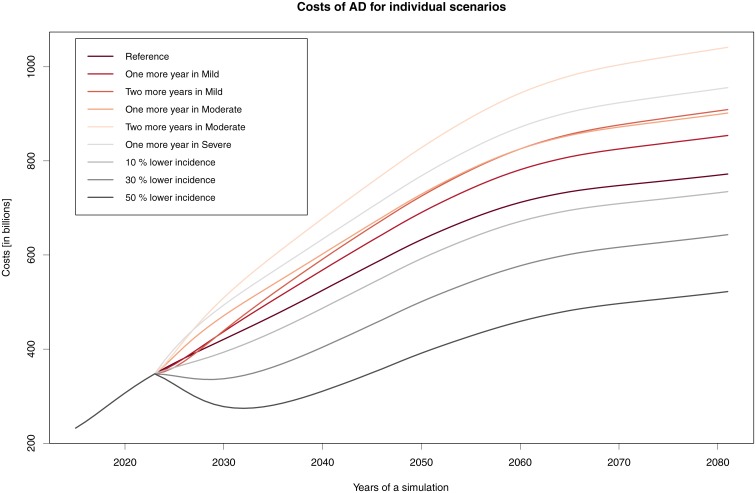
Cost of AD for individual scenarios. Change in total costs when introducing drugs and their different effects.

In general, we can say that no simulated scenario of treatment for prolonging the stay in a specific AD stage brings about savings. In all scenarios, there is an increase in costs due to two factors—namely, demographic development and prolonging the lives of those afflicted by AD (Fig 4).

In the most economically demanding option (i.e. prolongation in the Moderate stage by two years), we can expect that treatment and care costs will be €509 billion in 2030, €828 billion in 2050, and as much as €1 038 billion in 2080. In the least expensive option (i.e. prolongation in the Mild stage by two years), the costs will be €439 billion in 2030, €725 billion in 2050, and €906 billion in 2080.

When comparing these values to those in the literature, it is clear that further increases in expected costs can be anticipated. Wimo et al. [31] estimated an increase across the whole of Europe of about 43% between 2008 and 2030; this translates into an absolute value exceeding €250 billion. The costs of dementia in 2010 and 2015, according to the World Alzheimer Report (2015) [32].

**Fig 5:**
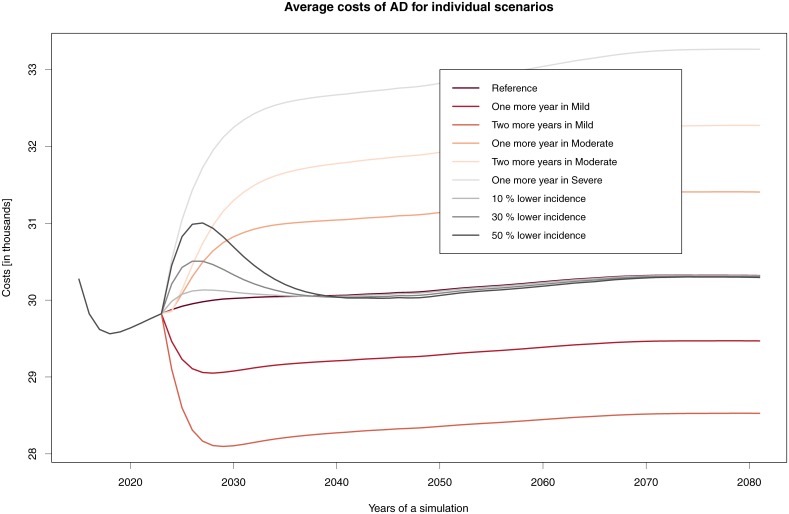
Average costs per patient when introducing drugs and considering their efficacy.

Fig 5 describes the development of average costs while disregarding population ageing; here, growth in the number of people in this category fundamentally affects total costs. The average costs are most favourable when a drug that prolongs the Mild stage by two years is developed: in this case, per-patient costs can drop to €28,097 in 2029. Support for staying in the Severe stage is the worst option, with the average per-patient cost in 2080 being almost €33,266.

On the other hand, in the remaining scenarios, it is assumed that people will not progress in the disease itself. In the modelled situation, the incidence rate was lowered by 10%, 30%, or 50%, which resulted in corresponding costs savings (see in Table 7 and Fig 4). This assumption also aligns with current research on diagnostics for the possible onset of AD, and to global preventive measures. Any treatment that prolongs one’s stay in a milder disease stage will improve quality of life among patients and caregivers alike, given projected demographic scenarios. Nevertheless, there will be no savings with regard to public spending. The amount of future costs remains in question, as they are fundamentally affected by the modelling approach taken.

For example, in [30], the authors reduce the annual rate of transition by a given percentage; compare this to the current approach, wherein the rate of transitions is derived based on a desired change to the length of stay in a specific AD stage.

Additionally, the current study differs from the literature in terms of the assumed death rate among AD patients. Anderson et al. [30] used a progressive death rate corresponding to a patient’s age and the two most advanced disease stages (i.e. Moderate and Severe).

The approach to prolonging one’s stay in a given disease stage would also affect the simulation results. The question is whether the treatment should have an effect on the probability of death and of staying in the same disease stage. In our simulations, the treatment changed only the probability of transition among stages (i.e. staying in the Mild or Moderate stage versus transitioning to a milder or worse stage): it did not change the probability of death (except in the Severe stage, where death is the only option, so to speak). Another possibility is changing the probability of transition to change also the probability of death, to simulate the fact that the treatment also improves quality of life. Both approaches can preserve the amount of time patients spend in other stages (i.e. not those that are prolonged), as is the case in the current study. However, following consultation with an expert on treatment developments, it became apparent that it is also possible that a treatment can affect not just one stage but also all the remaining stages (e.g. by prolonging the Mild stage for one year, the Moderate and Severe stages would also last longer).

All the aforementioned possibilities are supported by specialists in AD drug development, and they do appear to be realistic, according to current research and development results.

When interpreting the aforementioned models, we must consider certain risks that can distort the results. The first of these is that the calculated economic burden is based on two studies conducted in Europe. We could refine the calculation by undertaking further research; we could also perform meta-analysis at the beginning and then use the results as model inputs. However, given the unequivocal development of the economic burden in the years to come, we believe that the results would remain materially unchanged. Another limit of the current study is the lack of sufficient data pertaining to the actual number of AD patients. In all existing data sources, numbers are mere estimates, as many patients have not been properly diagnosed. Similarly, there is insufficient information on the AD incidence rate. Furthermore, the probability of death is based on studies that take into account only the stage of the patient’s disease, and not his or her age; in extending this research, it would be fitting to consider both of these factors. Simulations of drug developments look to understand the effects of prolonging a patient’s stay in the Mild or Moderate stages. The likelihood of death during these stages has been maintained, and the likelihood of transition to subsequent stages has changed. Only in the Severe stage can the simulation reduce the probability of death, because it is not possible to increase the probability of staying in this stage.

Furthermore, the monetarization of societal costs on the treatment of dementia is complicated by some variables, including disease–specific factors and macroeconomic factors. The macro factors concern in particular economic development, while the dementia–specific elements cover possibilities such as the development of more effective medications, changes in health care systems, or changes in prevalence and incidence of the disease. [33] Another problem is explained by the global imbalance between the prevalence of dementia and the costs allocated to dementia patient care. The highest societal costs per capita are in high–income countries (HIC) (89% of global expenditure), although the highest number of patients occurs in low- and mid–income countries (LMIC). Average wages used to estimate informal care costs are much lower in LMIC, this has a significant impact on total comparative costs which should be taken into account. [33] Last but not least, another volatile economic aspect is the question of discount rates. There is no general rule regarding discounts in healthcare. Some authors recommend discount rates ranging between 3 and 5 percent; [19, 34] others suggest a range between 2.5 and 8 percent. [35] Discount rates are expected to increase over time.

Some of the aforementioned factors that may distort the study results will be addressed in future research.

## Conclusion

Many European Commission regulations aim to improve quality of life among the ageing population, and to ensure a sufficient level of health and social services provision. In 2009, the European Initiative on Alzheimer’s Disease and Other Forms of Dementia was approved. Among individual European countries, time-tested practices have been obtained from research to help diagnose and treat disease, or to fund therapies.

Based on the model results, we can say that the development of new and more efficient drugs that prolong a patient’s stay in earlier stages of Alzheimer’s disease can improve his or her life and that of their caregivers. However, such drug developments will not help reduce the associated economic burden. If, at the very least, the current level of care is to be ensured, it will be essential to assume a continuous increase in expenditures. The only way to reduce spending will be to develop solutions that can be enacted prior to the onset of the illness.
